# Prognostic Potential of Secreted Modular Calcium-Binding Protein 1 in Low-Grade Glioma

**DOI:** 10.3389/fmolb.2021.666623

**Published:** 2021-11-19

**Authors:** Jing Wang, Shu Xia, Jing Zhao, Chen Gong, Qingsong Xi, Wei Sun

**Affiliations:** Department of Oncology, Tongji Hospital, Tongji Medical College, Huazhong University of Science and Technology, Wuhan, China

**Keywords:** SMOC1, low-grade glioma, prognostic value, biological function, tumor microenvironment

## Abstract

**Background:** Secreted modular calcium-binding protein 1 (SMOC1) belongs to a family of matricellular proteins; it was involved in embryo development, endothelial cell proliferation, angiogenesis, integrin–matrix interactions, cell adhesion, and regulation of glucose metabolism. Previous studies showed that the expression of SMOC1 was increased in some tumors. However, the prognostic value and the biological function of SMOC1 in tumor remain unclear.

**Methods:** In this study, we explored the expression profile and prognostic value of SMOC1 in pan-cancers, especially glioma, *via* multiple databases, including Oncomine, Gene Expression Profiling Interactive 2, PrognoScan, Kaplan–Meier plotter, and the Chinese Glioma Genome Atlas database. Furthermore, LinkedOmics was used to identify the genes coexpressed with SMOC1 and to perform Kyoto Encyclopedia of Genes and Genomes pathways and Gene Ontology analysis in low-grade glioma (LGG). Also, the Cancer Single-Cell State Atlas database was used to evaluate the correlation between SMOC1 expression and functional state activities in glioma cells. In addition, the Tumor Immune Estimation Resource and TISIDB databases were used to evaluate the correlations between SMOC1 expression and tumor-infiltrating immune cells in the tumor microenvironment.

**Results:** Compared with normal brain tissues, the expression of SMOC1 was increased in LGG tissues. The higher expression of SMOC1 was significantly correlated with better survival of LGG patients. Additionally, functional analyses showed that the SMOC1 coexpressed genes were inhibited in processes such as response to type I interferon and interferon-gamma, lymphocyte-mediated immunity, leukocyte migration, adaptive immune response, neutrophil-mediated immunity, T cell activation, and pathways including EMC–receptor interaction, Th17 cell differentiation, and leukocyte *trans*-endothelial migration in LGG. Moreover, the expression of SMOC1 was correlated with stemness, hypoxia, EMT, and metastasis of glioma cells. Additionally, the expression of SMOC1 expression was negatively correlated with levels of infiltrating B cells, CD8^+^ T cells, CD4^+^ T cells, macrophages, neutrophils and dendritic cells, and gene markers of most immune cells in LGG.

**Conclusion:** Our results suggest that SMOC1 could be a potential biomarker to determine prognosis and might play a specific role in the tumor microenvironment of glioma, thereby influencing the development and progression of glioma. These findings provide some new insights for further investigation.

## Introduction

For the past century, the classification of tumors has been based largely on the concept that tumors can be classified according to their histological features under a light microscope. Over the past 10 years, studies have clarified the genetic basis of tumorigenesis in various tumors, and such understanding raises the possibility that it may contribute to the further classification of tumors (Louis, 2012). Also, a better understanding and identification of predictive and prognostic biomarkers are needed for individualized treatment and improved survival.

Secreted modular calcium-binding protein 1 (SMOC1) was first isolated in 2002, which encodes a secreted modular glycoprotein (Vannahme et al., 2002). SMOC1 contains a follistatin-like domain, an EF-hand calcium-biding domain, two thyroglobulin-like domains, and a unique domain and is usually localized in the basement membrane of different tissues and also can present in other extracellular matrices (Vannahme et al., 2002). It belongs to a family of matricellular proteins that also include basement membrane-40 (also known as secreted protein acidic and rich in cysteine), as well as SMOC2 (Bornstein and Sage, 2002). SMOC2 was involved in multiple biological processes, including cell cycle progression (Liu et al., 2008), cell attachment (Maier et al., 2008), and tumor development (Su et al., 2016; Huang et al., 2017). Also, in previous studies, the expression of SMOC2 was decreased in gallbladder cancer (Gu et al., 2015) and advanced breast cancer (Fidalgo et al., 2015) but increased in metastatic head and neck squamous cell carcinoma (Hyakusoku et al., 2016). In endometrial carcinoma, SMOC2 was able to inhibit cell proliferation and overcome chemoresistance (Lu et al., 2019). SMOC1 has similar domains to SMOC2, except for its own unique domain (Vannahme et al., 2003). We assume that similar to SMOC2, SMOC1 might also be involved in tumor development. The expression of SMOC1 was increased in some brain tumors, such as oligodendroglioma (Brellier et al., 2011) and astrocytic tumors (Boon et al., 2004). Moreover, SMOC1 was identified as a new cancer-related protein by interacting with tenascin-c, which was an ECM protein and was highly expressed in many human cancers (Brellier et al., 2011). In U87 glioma cells, SMOC1 inhibits the tenascin-c-induced chemo-attractive effect (Brellier et al., 2011). Previous studies indicated that SMOC1 was involved in embryogenesis (Gersdorff et al., 2006), endothelial cell proliferation, angiogenesis (Awwad et al., 2015), integrin–matrix interactions, cell adhesion (Klemencic et al., 2013), and glucose metabolism (Montgomery et al., 2020). However, the role of SMOC1 in tumor genesis and progression is still unclear.

In this study, we aimed to systematically explore the gene expression and evaluate the prognostic values of SMOC1 in different cancers and uncover the potential functions of SMOC1 in specific cancers. The findings explored the prognostic value of SMOC1 in low-grade glioma (LGG) and provided new ideas for exploring the potential mechanism of SMOC1 in glioma development and progression.

## Methods

### Oncomine Database Analysis

The expression levels of SMOC1 messenger RNA (mRNA) in different types of tumors were analyzed in the Oncomine database (https://www.oncomine.org) (Rhodes et al., 2007). The threshold was determined according to the following values: gene ranking of top 10%, a *p*-value of 0.05, and fold change of 2.

### Tumor Immune Estimation Resource Analysis

The Tumor Immune Estimation Resource (TIMER, https://cistrome.shinyapps.io/timer/) (Li et al., 2017) was used to explore the expression of SMOC1 in various types of cancers and to evaluate the correlation between SMOC1 expression and the infiltrating level of immune cells, including B cell, CD8^+^ T cell, CD4^+^ T cell, macrophage, neutrophil, and dendritic cell. In addition, the correlation between SMOC1 expression and tumor-infiltrating immune cell gene markers was also explored through the correlation modules in the TIMER database. The gene markers were selected according to a previous report (Danaher et al., 2017), the CellMarker database (http://biocc.hrbmu.edu.cn/CellMarker/index.jsp) (Zhang X. et al., 2019), and the website of R&D Systems (https://www.rndsystems.com/cn/resources/cell-markers/immune-cells), which include markers of B cells, CD8^+^ T cells, T cells, follicular helper T cells (Tfh), T helper cells (Th1, Th2, and Th17), regulatory T cells (Treg), exhausted T cells, monocytes, TAMs, M1 macrophages, M2 macrophages, neutrophils, DCs, natural killer cells (NKs), and Mast cells. *p* < 0.05 was considered as statistically significant.

### PrognoScan Database Analysis

The PrognoScan database (http://dna00.bio.kyutech.ac.jp/PrognoScan/index.html) (Mizuno et al., 2009) was used to investigate the association between SMOC1 expression and survival in different types of tumors. To evaluate the prognostic value of SMOC1, the Cox *p*-value and hazard ratio (HR) with 95% confidence intervals (95% CIs) were calculated and displayed.

### Gene Expression Profiling Interactive Two Analysis

The Gene Expression Profiling Interactive 2 (GEPIA2, http://gepia2.cancer-pku.cn/#index) (Tang et al., 2017) was used to analyze the correlations between SMOC1 expression and patient prognosis in various types of tumors, as well as the expression of SMOC1 in LGG and glioblastoma (GBM). Furthermore, the correlations between gene SMOC1 and selected gene markers of tumor-infiltrating immune cells were also established *via* the GEPIA2 database. *p* < 0.05 was considered as statistically significant.

### Kaplan–Meier Plotter Analysis

The Kaplan–Meier Plotter (http://kmplot.com/analysis/index.php?p=service&cancer=lung) (Nagy et al., 2018) was used to evaluate the correlations between SMOC1 expression and survival of lung adenocarcinoma (LUAD) patients in lung cancer dataset. The HR with 95% CIs, log-rank *p*-value, and survival curve were calculated and displayed.

### Chinese Glioma Genome Atlas Database Analysis

The Chinese Glioma Genome Atlas (CGGA, http://www.cgga.org.cn/) (Yan et al., 2012) was used to analyze the expression of SMOC1 in different grades and subtypes of glioma and to perform survival analysis in specific glioma subtype. The HR with 95% CIs and log-rank *p*-value were calculated.

### Cancer Single-Cell State Atlas

The Cancer Single-Cell State Atlas (CancerSEA, http://biocc.hrbmu.edu.cn/CancerSEA/) (Yuan et al., 2019) database was used to evaluate the correlation between SMOC1 expression and functional state of various tumor cells. Significant correlations between gene expression and functional state activities were identified using Spearman's rank correlation test (correlation > 0.3) and Benjamini and Hochberg's false discovery rate (<0.05).

### TISIDB

TISIDB (http://cis.hku.hk/TISIDB/) (Ru et al., 2019) was used to verify the association of SMOC1 expression and the abundance of tumor-infiltrating lymphocytes. Spearman's test was performed to measure the correlations between SMOC1 expression and tumor-infiltrating lymphocytes. *p* < 0.05 was considered as statistically significant.

### LinkedOmics Database

The LinkedOmics database (http://www.linkedomics.org/login.php) (Vasaikar et al., 2018) was applied to identify SMOC1 coexpressed genes in The Cancer Genome Atlas (TCGA)–LGG cohort. Pearson's correlation coefficients were used to analyze the results and presented in volcano plots and heat maps. Gene set enrichment analysis (GSEA) tool was used to perform Kyoto Encyclopedia of Genes and Genomes (KEGG) pathways and Gene Ontology analysis, enrichment with 500 simulations and a minimum number of three genes.

## Results

### Expression Levels of Secreted Modular Calcium-Binding Protein 1 in Different Type of Tumors

**FIGURE 1:**
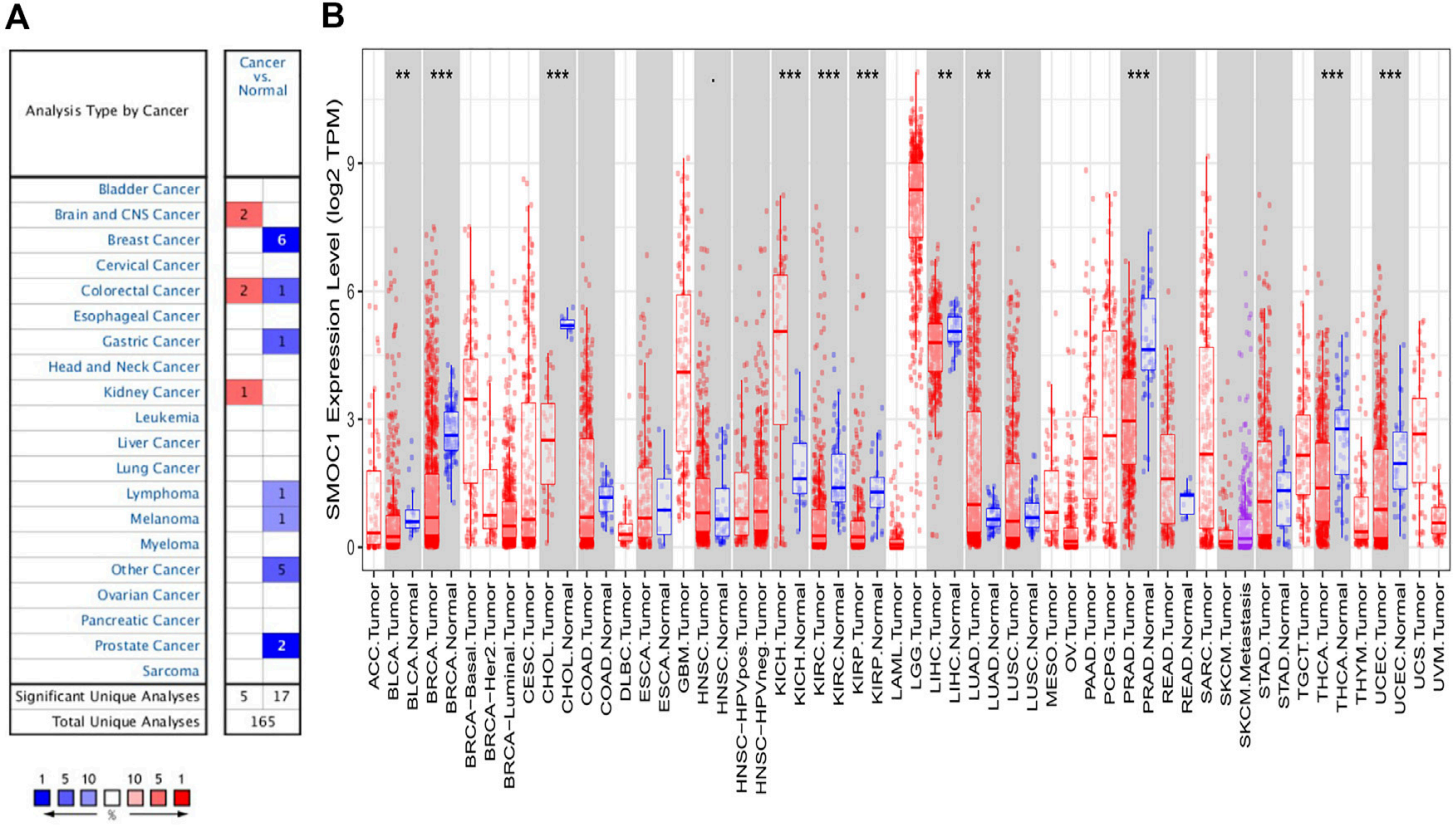
The expression levels of SMOC1 in different type of tumors. **(A)** The expression levels of SMOC1 in different type of tumors and normal tissues in Oncomine database. **(B)** The expression levels of SMOC1 in different type of tumors and adjacent normal tissues in TIMER database. (*p*-value < 0.1; *: *p*-value < 0.05; **: *p*-value < 0.01; ***: *p*-value < 0.001).

To preliminarily evaluate the role of SMOC1 in tumor genesis, the expression levels of SMOC1 mRNA in different types of tumors and normal tissue samples were analyzed in the Oncomine database. The expression levels of SMOC1 were higher in the kidney, brain, and central nervous system (CNS) cancers compared with the corresponding normal tissues (Figure 1A). Meanwhile, lower expression of SMOC1 was observed in melanoma, breast, colorectal, gastric, prostate, and other cancers. The details of SMOC1 expression in different types of tumors are listed in Supplementary Table S1. To further explore the expression of SMOC1 in pan-cancers, we analyzed the RNA-seq data from TCGA using the TIMER database. The differential expression patterns of SMOC1 in tumors and adjacent normal tissues are shown in Figure 1B. Obviously, SMOC1 expression was significantly downregulated in bladder urothelial carcinoma, breast invasive carcinoma (BRCA), cholangiocarcinoma, kidney renal clear cell carcinoma, kidney renal papillary cell carcinoma, liver hepatocellular carcinoma, prostate adenocarcinoma, thyroid carcinoma, and uterine corpus endometrial carcinoma, whereas the expression of SMOC1 was increased in kidney chromophobe and LUAD. Due to the lack of adjacent normal tissue, the changes of SMOC1 expression in cancers such as LGG and GBM were unable to show.

### Prognostic Value of Secreted Modular Calcium-Binding Protein 1 Expression in Different Type of Cancers

To evaluate the prognostic value of SMOC1 in different types of cancers, we analyzed the correlations between SMOC1 expression and survival of human cancers in different databases. First, we examined the effect of SMOC1 expression on cancer survival in PrognoScan and listed the full results in Supplementary Table S2. The expression of SMOC1 was significantly correlated with the survival of six cancer types, including brain glioma, colorectal cancer, eye uveal melanoma, breast cancer, LUAD, and ovarian cancer (Table 1). Compared with low SMOC1 expression, high expression level of SMOC1 was correlated with better prognosis in brain glioma [overall survival (OS), HR = 0.55, 95% CIs, 0.4–0.75, Cox *p*-value = 0.0001], breast cancer, and ovarian cancer. However, high expression of SMOC1 was associated with poor prognosis in colorectal cancer and LUAD.

**TABLE 1:** Prognostic value of SMOC1 in different types of tumors in PrognoScan database.

Dataset	Cancer type	Subtype	Endpoint	Cohort	N	Cox *p*-VALUE	ln (HR)	HR [95% CI-low CI-upp]	
**GSE4412-GPL97**	Brain cancer	Glioma	Overall Survival	UCLA (1996–2003)	74	0.000141867	-0.592464	0.55 [0.41–0.75]
**GSE4412-GPL97**	Brain cancer	Glioma	Overall Survival	UCLA (1996–2003)	74	0.00106869	-0.281186	0.75 [0.64–0.89]
**GSE14333**	Colorectal cancer		Disease Free Survival	Melbourne	226	0.00834547	0.263706	1.30 [1.07–1.58]
**GSE17537**	Colorectal cancer		Overall Survival	VMC	55	0.0143359	1.39887	4.05 [1.32–12.41]
**GSE14333**	Colorectal cancer		Disease Free Survival	Melbourne	226	0.0147715	0.24837	1.28 [1.05–1.57]
**GSE22138**	Eye cancer	Uveal melanoma	Distant Metastasis Free Survival	BRCIC	63	0.0151028	-32.3066	0.00 [0.00–0.00]
**GSE17536**	Colorectal cancer		Disease Free Survival	MCC	145	0.0178348	0.631362	1.88 [1.12–3.17]
**GSE9893**	Breast cancer		Overall Survival	Montpellier, Bordeaux, Turin (1989–2001)	155	0.0308433	-0.56531	0.57 [0.34–0.95]
**GSE17536**	Colorectal cancer		Disease Free Survival	MCC	145	0.0374849	0.987085	2.68 [1.06–6.80]
**GSE31210**	Lung cancer	Adenocarcinoma	Overall Survival	NCCRI	204	0.0377567	0.36064	1.43 [1.02–2.02]
**GSE1378**	Breast cancer		Relapse Free Survival	MGH (1987–2000)	60	0.038728	0.282083	1.33 [1.01–1.73]
**GSE17260**	Ovarian cancer		Progression Free Survival	Niigata (1997–2008)	110	0.0400779	-0.513388	0.60 [0.37–0.98]
**GSE9195**	Breast cancer		Relapse Free Survival	GUYT2	77	0.0423734	1.23944	3.45 [1.04–11.43]

Note: This table only shows those of significant difference (Cox *p* < 0.05).

**FIGURE 2:**
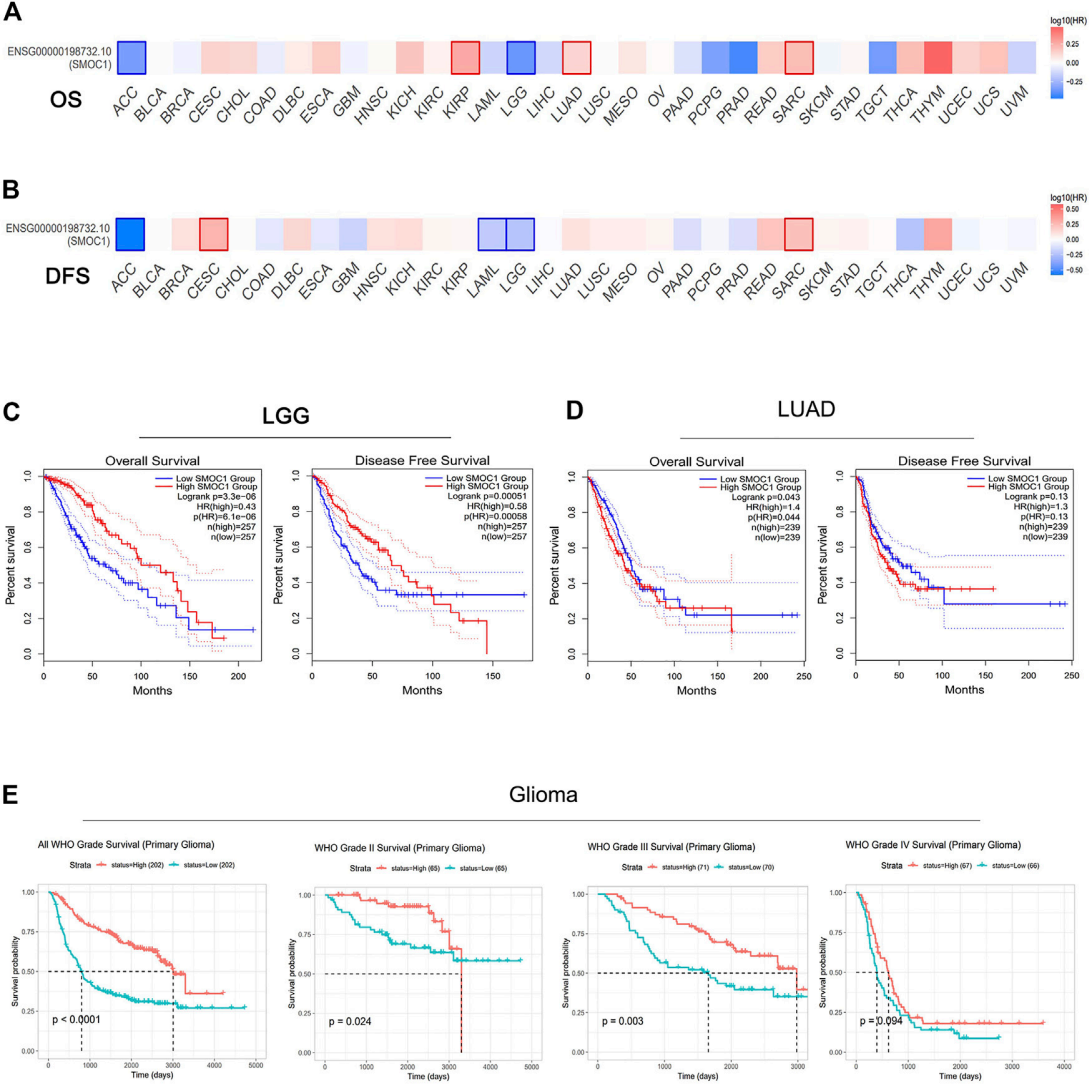
Prognostic value of SMOC1 expression in different type of tumors. **(A,B)** Survival heat map of SMOC1 in 33 TCGA cancer types in GEPIA2. The HRs of SMOC1 were shown in logarithmic scale (log10) in the heat map. The red and blue blocks represent higher and lower risks, respectively. The significant results in prognostic analysis were marked with framed rectangles. OS and DFS survival curves of **(C)** LGG and **(D)** LUAD in GEPIA2. **(E)** Survival probability of primary gliomas (all WHO Grade, WHO Grade II, Grade III and Grade IV) in CGGA database. (HR, hazard ratios; OS, overall survival; DFS, disease free survival).

Then, we further evaluated the relationships between SMOC1 expression and prognosis in 33 cancer types from the TCGA project in GEPIA2. The impact of SMOC1 expression on the survival of these 33 cancer types is presented in Figure 2A (OS) and **2B** (disease-free survival). Notably, survival of LGG and LUAD were also significantly correlated with SMOC1 expression. Higher expression of SMOC1 was correlated with better survival of LGG (OS, HR = 0.43, *p*-value = 3.3e-06; disease-free survival, HR = 0.58, *p*-value = 0.0005) (Figure 2C) but worse survival of LUAD (OS, HR = 1.4, *p*-value = 0.043) (Figure 2D). No correlation was observed between SMOC1 expression and the survival probability of GBM (Supplementary Figure S1A).

We then used Kaplan–Meier plotter database to further examine the prognostic value of SMOC1 in LUAD. High expression of SMOC1 was correlated with poor prognosis of LUAD (progression-free survival, HR = 1.5, *p*-value = 0.014) but was not related to OS in LUAD (Supplementary Figure S1B). The CGGA database was used as an independent database to confirm the prognostic value of SMOC1 in glioma. Consist with GEPIA2 analysis, a significant positive correlation was found between SMOC1 expression and better prognosis of all World Health Organization (WHO) grade I (*p* < 0.0001), WHO grade II (*p* = 0.024), and WHO grade III (*p* = 0.003) glioma, but no correlation was observed between the expression of SMOC1 and WHO grade IV glioma (*p* = 0.094) (Figure 2E).

### Expressions of Secreted Modular Calcium-Binding Protein 1 in Glioma and Lung Adenocarcinoma

**FIGURE 3:**
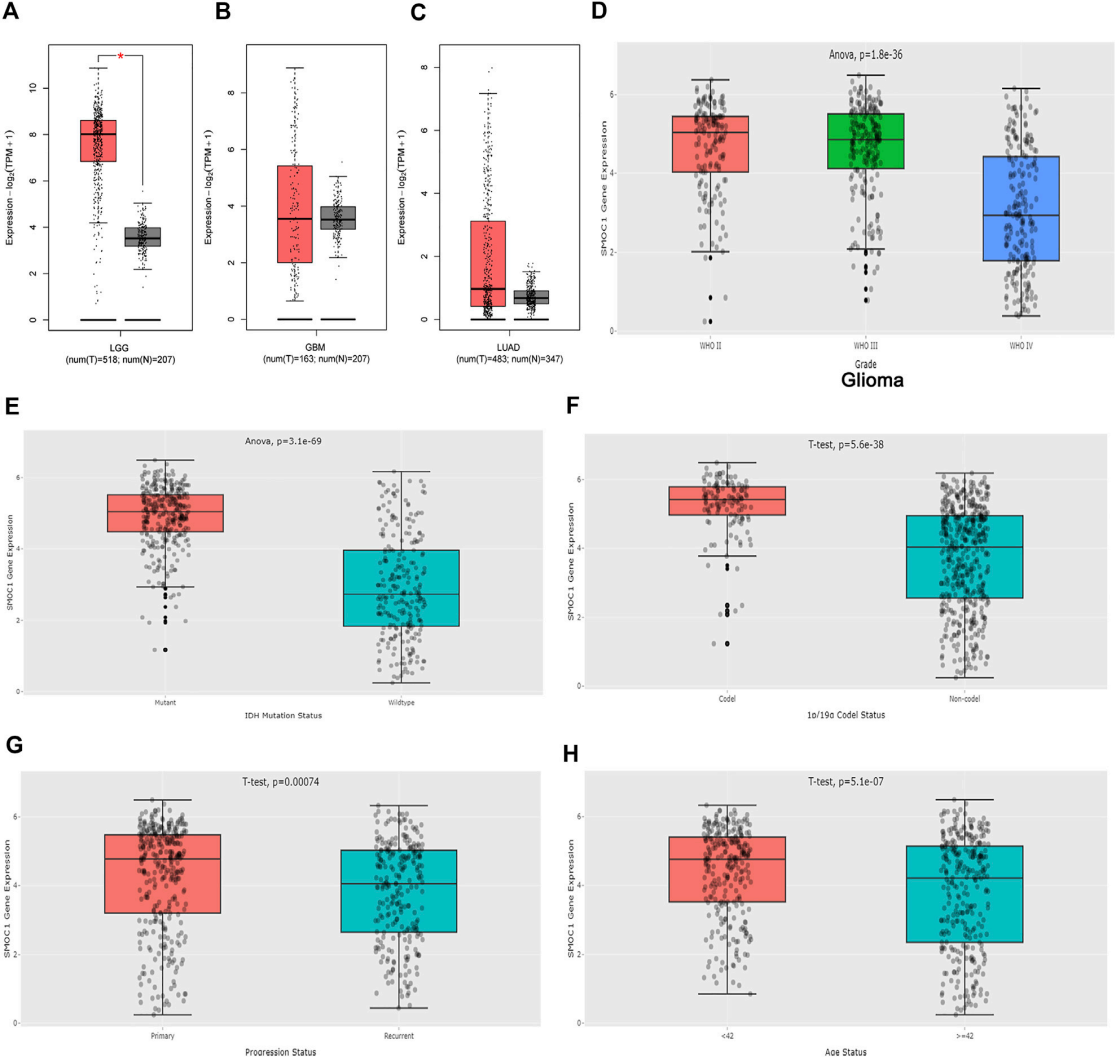
Expression of SMOC1 in Glioma and LUAD. The expression of SMOC1 in LGG **(A)**, GBM **(B)** and LUAD **(C)** analyzed by GEPIA2. Red box: tumor samples from TCGA, grey box: paired normal tissues from TCGA and GTEx. The expression of SMOC1 in different grade **(D)** and subtype **(E–H)** of Glioma analyzed by CGGA.

The expressions of SMOC1 in glioma (LGG and GBM) and LUAD were further analyzed in the GEPIA2 database. The expression of SMOC1 was significantly increased in tumor samples of LGG (Figure 3A). When compared with normal tissues, GBM and LUAD samples showed no marked changes in SMOC1 expression levels (Figures 3B, C). Furthermore, we analyzed the expression of SMOC1 in the CGGA database. Compared with WHO grade IV glioma, SOMC1 expression was remarkably increased in WHO grade II and grade III glioma (*p* = 1.8e-36) (Figure 3D). We further studied the relationship between SMOC1 expression and LGG subtypes. Through the CGGA dataset, we explored the relationship between SMOC1 expression and glioma subtypes, including IDH mutant status, 1p19q codeletion status, recurrent status, and patient's age status. We found that the expression of SMOC1 was significantly increased in IDH mutant gliomas (*p* = 3.1e-69) (Figure 3E), 1p19q co-deletion gliomas (*p* = 5.6e-38) (Figure 3F), primary gliomas (*p* = 0.00074) (Figure 3G), and the age< 42 group (*p* = 5.1e-07) (Figure 3H).

### Genes Correlated With Secreted Modular Calcium-Binding Protein 1 in Low-Grade Glioma

**FIGURE 4:**
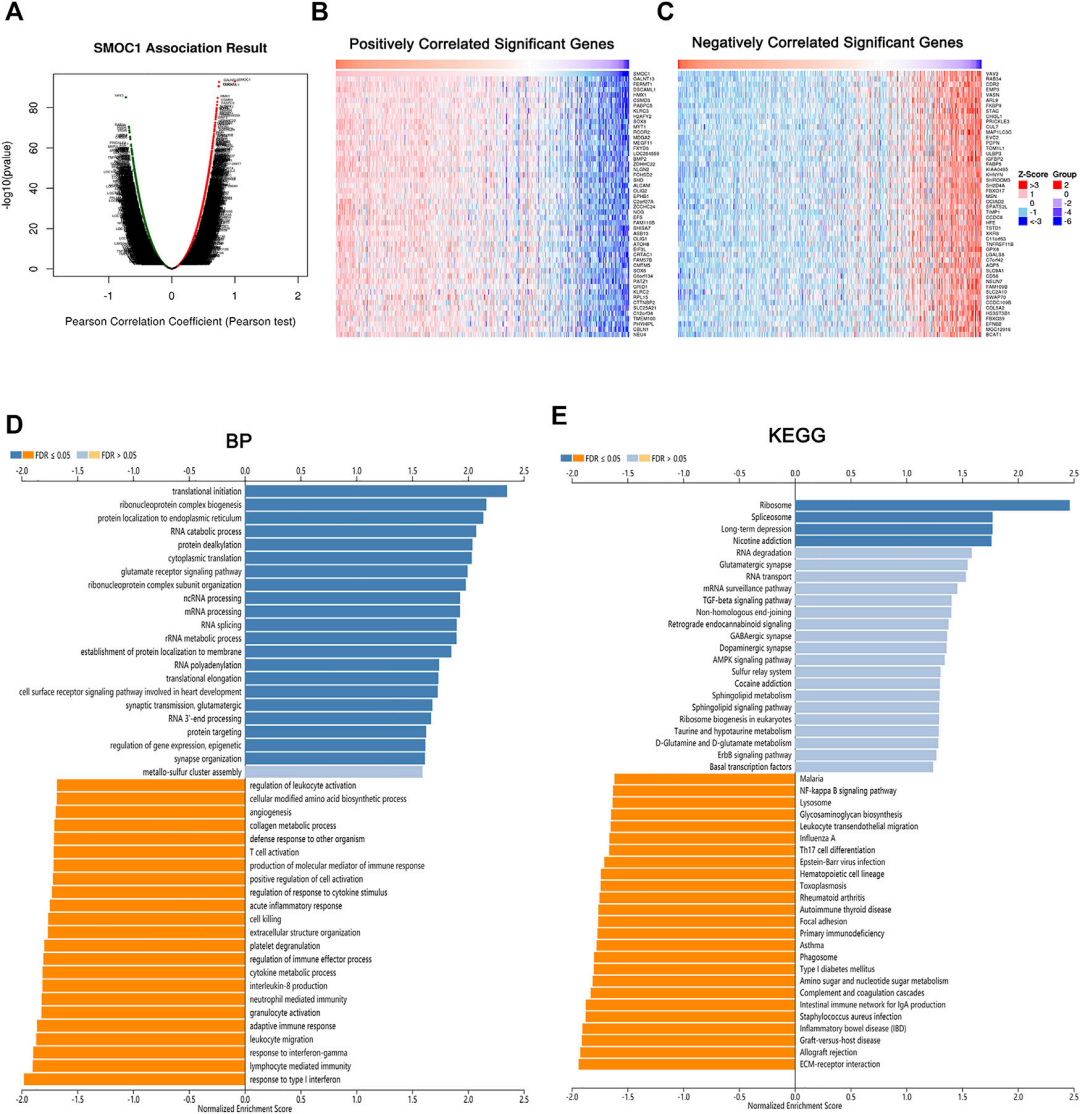
Genes correlated with SMOC1 in LGG. **(A)** SMOC1 correlated genes in LGG cohort identified by Pearson test. **(B,C)** The top 50 genes positively (red) and negatively (blue) correlated with SMOC1 in LGG were showing via heat map. **(D)** GO annotations and **(E)** KEGG pathways analysis of SMOC1 in LGG.

To explore the potential biological role of SMOC1 in LGG, we used the LinkedOmics database to identify the differentially expressed genes that were correlated with SMOC1. The identified genes that positively (red dots) and negatively (green dots) correlated with SMOC1 are shown in Figure 4A. Also, the heat maps present the top 50 positively (Figure 4B) and negatively (Figure 4C) correlated genes. GSEA tool was used to perform analyses of KEGG pathways and Gene Ontology functional enrichment of the differentially expressed genes. The results showed that the SMOC1 coexpressed genes were enriched for transnational initiation, ribonucleoprotein complex biogenesis, and protein localization, whereas the genes involved in processes such as response to type I interferon and interferon-gamma, lymphocyte-mediated immunity, leukocyte migration, adaptive immune response, neutrophil-mediated immunity, and T cell activation were inhibited in LGG (Figure 4D). The KEGG pathway analyses showed that the SMOC1 coexpressed genes were mainly enriched in pathways such as ribosome and spliceosome. The pathways such as EMC–receptor interaction, allograft rejection, graft-*versus*-host disease, Th17 cell differentiation, and leukocyte *trans*-endothelial migration were inhibited in LGG (Figure 4E).

### Correlations Between Secreted Modular Calcium-Binding Protein 1 Expression and Functional State Activities in Glioma Cells

**FIGURE 5:**
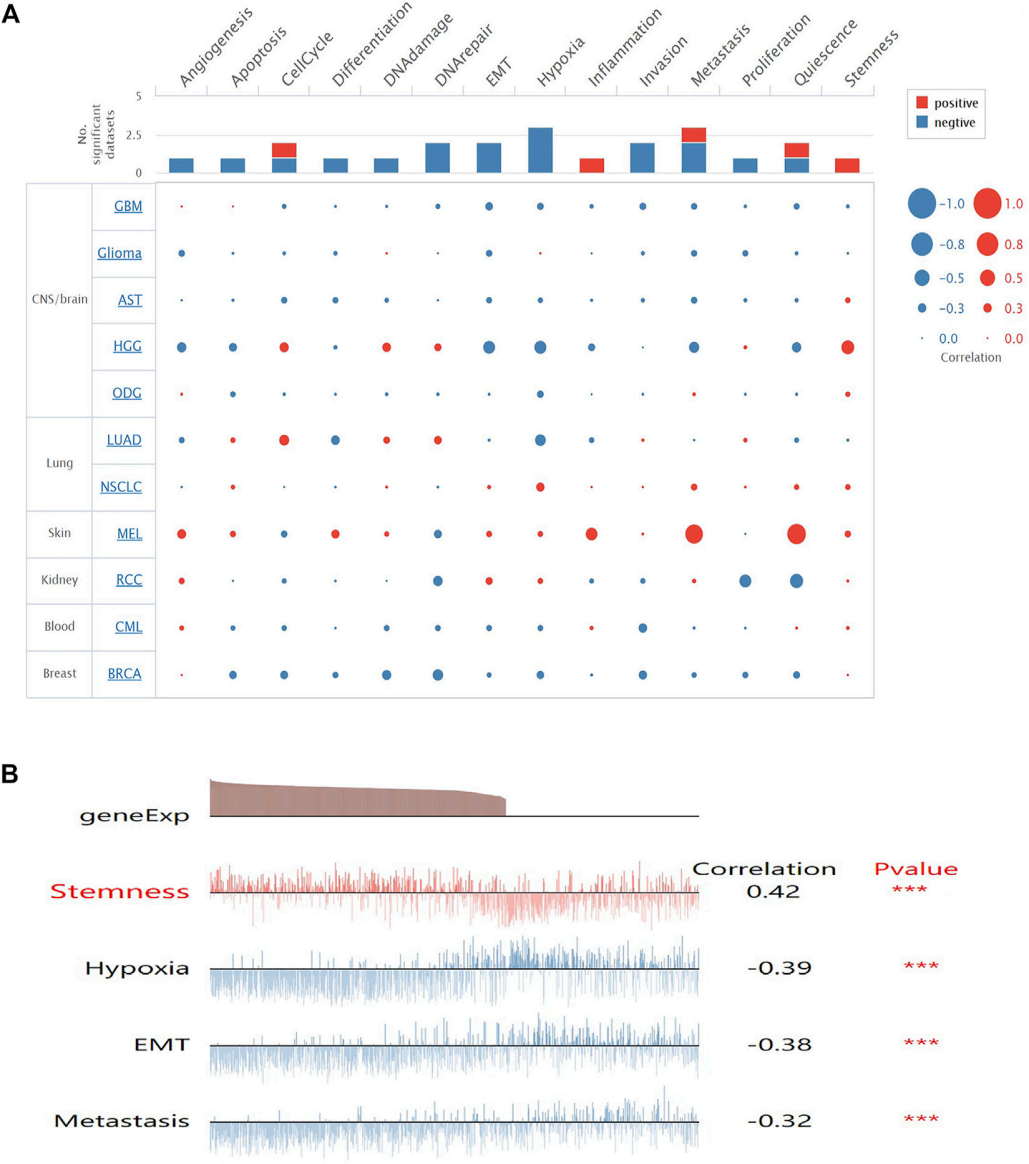
Correlations between SMOC1 expression and functional state activities in glioma cells. **(A)** The relevance of SMOC1 expression and functional state activities in different type of tumors. The size of plots indicates the strength of correlation, red indicates positive correlation, and blue indicates negative correlation, **(B)** Details of correlations between SMOC1 expression and functional state activities in HGG. ***p<0.001.

To get a better understanding of the underlying mechanisms of SMOC1 in glioma, we analyzed the correlations between SMOC1 expression and functional state activities across various types of tumors in the CancerSEA database. The expression of SMOC1 has been analyzed at the single-cell level of different types of tumors (Figure 5A), including CNS/brain tumors (GBM, glioma, aspartate aminotransferase, high-grade glioma, and oligodendroglioma), lung cancer (LUAD and nonsmall cell lung cancer), skin melanoma, renal cell carcinoma, chronic myelogenous leukemia, and BRCA. The expression of SMOC1 was associated with various functional states in different types of glioma cells (Figure 5A). In high-grade glioma, SMOC1 was significantly positively correlated with stemness (correlation = 0.42, *p* < 0.001) and negatively associated with hypoxia (correlation = -0.39, *p* < 0.001), EMT (correlation = -0.38, *p* < 0.001), and metastasis (correlation = -0.32, *p* < 0.001) (Figure 5B).

### Correlations Between Secreted Modular Calcium-Binding Protein 1 Expression and Immune Infiltration in Glioma

**FIGURE 6:**
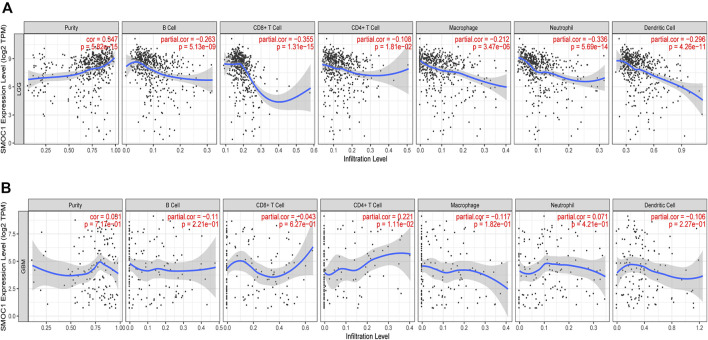
Correlations between SMOC1 expression and immune infiltration in Glioma. The correlation between SMOC1 expression and the immune infiltrations of tumor purity, B cell, CD8+ T cell, CD4+ T cell, Macrophage, Neutrophil, and Dendritic cell in LGG **(A)** and GBM **(B)**.

The TIMER database was used to investigate the relationship between immune infiltration and SMOC1 expression in glioma. We found that SMOC1 was positively correlated with tumor purity (cor = 0.347, *p* = 5.82e-15) in LGG but not in GBM. The expression of SMOC1 was significantly negatively correlated with levels of infiltrating B cells (cor = −0.263, *p* = 5.13e-09), CD8^+^ T cells (cor = −0.355, *p* = 1.31e-15), CD4^+^ T cells (cor = −0.108, *p* = 1.81e-02), macrophages (cor = −0.212, *p* = 3.47e-06), neutrophils (cor = -336, *p* = 5.69e-14), and dendritic cells (cor = −0.296, *p* = 4.26e-11) in LGG tumor microenvironment (Figure 6A). However, there was no correlation between SMOC1 expression and levels of infiltrating immune cells in GBM, except for CD4^+^ T cells (cor = 0.221, *p* = 1.11e-02) (Figure 6B). Similar results were also observed in the TISIDB analysis (Supplementary Figure S2).

### Correlations Between Secreted Modular Calcium-Binding Protein 1 and Gene Markers of Infiltrating Immune Cells in Glioma

To evaluate the more specific link between SMOC1 and tumor immune infiltration, we analyzed the correlations between SMOC1 and markers of various immune cells in LGG using the TIMER (Table 2) and GEPIA2 databases (Supplementary Table S3). Also, the correlations in GBM were analyzed as a control. According to previous research, we selected gene markers of different immune cell populations, including B cells, CD8^+^ T cells, T cells (general), monocytes, TAMs, M1 and M2 macrophages, neutrophils, DCs, NKs, mast cells, and different functional T cells, such as Tfh, Treg, exhausted T cells, Th1, Th2, and Th17. After adjusting for tumor purity, the expression of SMOC1 was significantly negatively correlated with gene markers of most immune cells in LGG, especially gene markers of CD8^+^ T cells, T cells (general), Th2 cells, dendritic cells, TAMs, and M1 macrophages (Table 2). However, there were only six gene markers significantly correlated with SMOC1 expression in GBM. The results from the GEPIA2 database (Supplementary Table S3) were similar to those of the TIMER analysis.

**TABLE 2:** Correlations between SMOC1 and gene markers of immune cells in LGG and GBM by TIMER.

Immune cell	Gene markers	LGG				GBM			
		None		Purity		None		Purity	
		Cor	*p* -value	Cor	*p* -value	Cor	*p* -value	Cor	*p* -value
B cell	*CD19*	−0.312	***	−0.273	***	0.011	0.892	−0.001	0.988
	*CD79A*	−0.023	0.596	−0.014	0.757	0.021	0.798	0.027	0.753
CD8^+^ T cell	CD8A	−0.462	***	-0.378	***	−0.025	0.762	−0.045	0.601
	*CD8B*	−0.253	***	−0.16	**	−0.009	0.912	−0.008	0.926
T cell (general)	CD3D	0.377	***	−0.32	***	−0.041	0.617	−0.037	0.664
	*CD3E*	−0.388	***	−0.345	***	−0.097	0.233	−0.109	0.206
	*CD2*	−0.389	***	−0.356	***	−0.053	0.515	−0.056	0.519
Tfh	*BCL6*	0.319	***	0.283	***	0.111	0.173	0.116	0.176
	*IL21*	−0.052	0.243	−0.063	0.167	-0.044	0.586	−0.037	0.666
Th1	*TBX21*	−0.165	**	−0.165	**	0.326	***	0.351	***
	*STAT4*	−0.292	***	−0.244	***	0.059	0.466	0.049	0.572
	*STAT1*	−0.293	***	−0.298	***	−0.016	0.841	−0.019	0.824
	*IFNG*	−0.216	***	−0.195	***	0.021	0.799	0.017	0.84
Th2	*STAT6*	−0.483	***	−0.386	***	0.114	0.161	0.189	0.027
	*GATA3*	−0.331	***	−0.309	***	−0.176	0.03	−0.17	0.047
	*STAT5A*	−0.348	***	−0.253	***	−0.167	0.039	−0.155	0.07
	*IL13*	0.015	0.74	0.011	0.803	0.119	0.143	0.1	0.248
Th17	*STAT3*	−0.103	0.019	−0.118	*	−0.133	0.102	−0.133	0.123
	*IL17A*	0.009	0.834	−0.002	0.958	−0.013	0.87	−0.015	0.862
Treg	FOXP3	0.102	0.02	0.099	0.031	0.002	0.984	0.008	0.923
	CCR8	−0.125	*	−0.13	*	−0.05	0.536	−0.056	0.515
	STAT5B	0.349	***	0.256	***	0.007	0.93	−0.004	0.96
	TGFB1	−0.209	***	−0.125	*	−0.151	0.063	−0.151	0.078
T cell exhaustion	PDCD1	−0.241	***	−0.204	***	0.076	0.349	0.081	0.342
	CTLA4	−0.285	***	−0.244	***	-0.006	0.943	−0.004	0.961
	LAG3	0.054	0.222	0.038	0.412	0.22	*	0.243	*
	*HAVCR2*	−0.284	***	−0.201	***	0.114	0.162	0.162	0.059
	*GZMB*	−0.269	***	−0.292	***	−0.135	0.097	−0.113	0.188
Monocyte	*CD86*	−0.253	***	−0.17	**	0.029	0.718	0.062	0.472
	*CSF1R*	−0.153	**	−0.038	0.411	0.108	0.183	0.14	0.102
TAM	*CD68*	−0.192	***	−0.132	*	0.058	0.472	0.122	0.157
	*CCL2*	−0.313	***	−0.266	***	−0.195	0.016	−0.196	0.022
	*CCL5*	−0.385	***	−0.345	***	−0.049	0.548	−0.028	0.745
M1	*NOS2*	−0.16	**	−0.128	*	−0.277	**	−0.277	*
	*IRF5*	−0.264	***	−0.172	**	0.17	0.035	0.224	*
	*PTGS2*	−0.245	***	−0.186	***	−0.107	0.186	−0.107	0.212
M2	*CD163*	−0.165	**	−0.164	**	−0.101	0.213	−0.105	0.223
	*VSIG4*	−0.085	0.054	−0.011	0.816	0.022	0.783	0.043	0.619
	*MS4A4A*	−0.065	0.138	−0.056	0.221	−0.022	0.782	−0.007	0.934
Neutrophil	*CEACAM8*	0.003	0.953	−0.005	0.917	0.04	0.62	0.06	0.484
	*ITGAM*	−0.279	***	−0.18	***	0.033	0.683	0.058	0.498
	*CCR7*	−0.211	***	−0.178	***	0.053	0.519	0.081	0.346
	*MPO*	−0.303	***	−0.221	***	0.187	0.021	0.204	0.017
DC	*CD1C*	0.093	0.035	−0.064	0.162	−0.068	0.401	−0.065	0.447
	*HLA-DPB1*	−0.423	***	−0.381	***	−0.03	0.711	−0.002	0.986
	*HLA-DQB1*	−0.38	***	−0.344	***	−0.1	0.218	−0.102	0.235
	*HLA-DRA*	−0.424	***	−0.389	***	−0.029	0.723	−0.028	0.749
	*HLA-DPA1*	−0.403	***	−0.366	***	−0.007	0.93	0.017	0.847
	*NRP1*	−0.11	0.012	−0.16	**	−0.188	0.02	−0.196	0.022
	*ITGAX*	−0.219	***	−0.115	0.012	0.137	0.091	0.183	0.033
NK cell	*KIR3DL1*	−0.057	0.194	−0.038	0.409	0.023	0.779	0.034	0.696
	*KIR2DL1*	−0.072	0.101	−0.087	0.059	0.034	0.674	0.07	0.415
	*KIR2DL3*	−0.207	***	−0.208	***	0.032	0.698	0.051	0.555
	*KIR2DL4*	−0.187	***	−0.182	***	0.011	0.896	−0.023	0.793
	*KIR3DL2*	−0.13	*	−0.128	*	−0.066	0.417	−0.057	0.512
	*KIR3DL3*	-0.015	0.74	−0.021	0.639	0.105	0.198	0.092	0.287
	*KIR2DS4*	−0.128	*	−0.095	0.039	−0.039	0.629	−0.029	0.736
Mast cell	*TPSB2*	−0.085	0.054	−0.093	0.042	0.182	0.025	0.191	0.026
	*CPA3*	−0.121	*	−0.158	**	0.15	0.064	0.144	0.093
	*HDC*	−0.398	***	−0.386	***	0.134	0.1	0.153	0.074

Note: None, correlation without adjustment; Purity, correlation adjusted by tumor purity; M1, M1 Macrophage; M2, M2 Macrophage; DC, Dendritic cell; NK cell: Natural killer cell; Cor, R value of Spearman’s correlation. *p*-value significant codes: *p < 0.01; **p < 0.001; ***p < 0.0001.

## Discussion

Gliomas are the most common malignant tumor of the CNS, with a character of aggressive growth, poor outcome of the patient, and high rates of recurrence (Wen and Kesari, 2008). According to the CBTRUS report, glioma represents approximately 25.1% of all primary and 80.8% of malignant tumors in the brain and other CNS tumors diagnosed in the United States in 2013–2017 (Ostrom et al., 2020). Also, the WHO classifies glioma into four grades: WHO grades I, II, III, and IV (Louis et al., 2016). LGG usually refers to gliomas other than WHO grade IV glioma (GBM) and accounts for approximately 10% of all primary brain tumors (Ostrom et al., 2020). The prognosis of gliomas varies widely by WHO classification. Compared with GBM patients, LGG patients usually have an indolent course and longer survival (Forst et al., 2014). However, despite initially growing slow, LGG can transform to GBM with time (Morshed et al., 2019). According to the previous study (Afra et al., 1999), nearly half of the patients with LGG will experience malignant transformation, usually within 5 years. However, the risk factors for the development of LGG are poorly understood at present, and the current understanding of the mechanism underlying the growth and invasion of glioma is limited. The application of IDH mutate-status in 2016 WHO Classification of CNS tumors showed a great significance of molecular diagnosis in glioma (Louis et al., 2016). However, more molecular biomarkers are still needed to be discovered.

SMOC1 was first reported in 2002; we know not much about the biological function of SMOC1. However, its potential importance cannot be ignored because it has been linked with embryogenesis (Gersdorff et al., 2006), osteoblast differentiation (Choi et al., 2010), and some forms of cancer. One *in silico* study analyzed the expression and prognostic data of long noncoding RNAs, microRNAs, and mRNAs in the colon cancer dataset from the TCGA database and identified five mRNAs, including SMOC1, as potential prognostic biomarkers for colon cancer (Huang and Pan, 2019). Besides, another study suggests that the aberrant expression of SMOC1 can inhibit the proliferation and colony formation in colorectal cancer cells, as well as tumor formation *in vivo* (Aoki et al., 2018). This suggests that SMOC1 may act as a tumor suppressor. A recent study has analyzed the methylation data of the glioma project from the TCGA database and identified 10 glioma grade-associated cytosine-phosphate guanine sites, which targeted four genes, including SMOC1 (Weng and Salazar, 2021). Another *in silico* study identified a seven-gene signature, including SMOC1, which was positively correlated with 5-years OS of glioma patients (Zhang G.-H. et al., 2019). However, the specific relationship between the expression level of SMOC1 and the prognosis of glioma patients has not been evaluated yet.

In this study, we first systematically analyzed the expression level of SMOC1 and its prognostic value in glioma. Compared with levels in normal tissue, the expression of SMOC1 was aberrantly in many cancers; it was reduced in bladder urothelial carcinoma, BRCA, cholangiocarcinoma, kidney renal clear cell carcinoma, kidney renal papillary cell carcinoma, liver hepatocellular carcinoma, prostate adenocarcinoma, thyroid carcinoma, and uterine corpus endometrial carcinoma but increased in kidney chromophobe, LUAD, and LGG. These data suggest that the alterations in SMOC1 expression depend on the type of cancer. Survival analyzes from PrognoScan and GEPIA2 showed that the expression level of SMOC1 was correlated with the prognosis of brain glioma (LGG) and LUAD patients. Therefore, we further analyzed the expression of SMOC1 in glioma and LUAD *via* the GEPIA2 and CGGA databases. The results showed that the expression of SMOC1 was remarkably increased in tumor samples of LGG, whereas there were no significant changes in GBM and LUAD samples, which was consistent with previous studies (Boon et al., 2004; Brellier et al., 2011). In addition, we found that the expression of SMOC1 was significantly increased in subtypes of glioma through the CGGA dataset. Compared with IDH-wild-type, 1p19q non-codeletion, and recurrent subtypes, the expression of SMOC1 was increased in IDH mutant, 1p19q co-deletion, and primary gliomas. In the age < 42 years group, a higher expression level of SMOC1 was also observed. As we know, IDH wild-type, 1p19q non-codeletion, recurrent gliomas, and age > 40 years were poor prognostic factors in LGG (Nabors et al., 2017). These results confirmed the upregulation of SMOC1 expression in LGG and suggested that the expression of SMOC1 increases with the decrease of tumor malignancy. Consistent with the analysis of the TCGA datasets in GEPIA2, the survival analysis of CGGA datasets also showed a significant positive correlation between SMOC1 expression and better prognosis in all WHO grade I, WHO grade II, and WHO grade III gliomas but not in WHO grade IV glioma. These findings further confirmed the prognostic value of SMOC1 in specific types of cancer. Therefore, based on the consistent results of the association between SMOC1 expression and survival of LGG patients, we have reason to believe that SMOC1 can serve as a good prognostic biomarker in LGG. As mentioned before, the expression of SMOC1 was increased in oligodendroglioma (Brellier et al., 2011) and astrocytic tumors (Boon et al., 2004). Also, SMOC1 has even been identified as a new cancer-related protein by interacting with tenascin-c, and it inhibits the tenascin-c induced chemo-attractive effect (Brellier et al., 2011) in U87 glioma cells. However, the function of SMOC1 in glioma is still unclear. Also, it is necessary to focus on its precise effects in this cancer type, as well as the underlying mechanisms.

According to previous studies, the gene SMOC1 encodes a secreted modular glycoprotein (Vannahme et al., 2002). This protein belongs to a family of matricellular proteins, and it was generally expressed on the basement membrane of different tissues in adult animals and also can be present in other extracellular matrices (Vannahme et al., 2002). During mouse development, SMOC1 was expressed in the basement membrane zones of the brain, blood vessels, lung, heart, and many other tissues. This broad distribution suggests that SMOC1 might have multifunctional roles during mouse embryogenesis (Gersdorff et al., 2006). Besides, previous studies have shown that SMOC1 might also involve in angiogenesis. SMOC1 was highly expressed in proliferation endothelial cells, and the expression of SMOC1 was regulated by inflammatory cytokines and nitric oxide (Dreieicher et al., 2009). Another study indicated that SMOC1 acts as a negative feedback regulator of the activin-like kinase 5 signal pathway by binging to endothelin, leading to activation of transforming growth factor-beta signal pathway and activin-like kinase 1, thus promoting endothelial cell proliferation and angiogenesis (Awwad et al., 2015). Moreover, one study reported that SMOC1 might be involved in integrin–matrix interactions and cell adhesion (Klemencic et al., 2013). In addition to its role in matrix remodeling, SMOC1, as a circulating glycoprotein, was identified as a regulator of glucose homeostasis (Montgomery et al., 2020). A recent study identified SMOC1 as a novel thrombin-activating protein, which enhances the action of thrombin *in vivo* and *in vitro* (Delgado Lagos et al., 2021).

In this study, with the GSEA tool of LinkedOmics database, we found that the SMOC1 coexpressed genes were mainly enriched for protein localization and in pathways such as ribosome in LGG, whereas the genes involved in processing such as response to lymphocyte- and neutrophil-mediated immunity, leukocyte migration, adaptive immune response, T cell activation and in pathways such as ECM–receptor interaction, Th17 cell differentiation, and leukocyte *trans*-endothelial migration were inhibited in LGG. In the further analysis in CancerSEA, we found that the expression of SMOC1 was correlated with several important functional states in glioma cells, especially stemness, hypoxia, EMT, and metastasis. These results strongly suggested that SMOC1 might influence various processes in the glioma tumor microenvironment. It is well known that interactions between malignant cells and the extracellular environment are critical for cancer development and progression (Rangarajan and Weinberg, 2003). Additionally, we observed in our analysis that there was a positive correlation between SMOC1 expression and tumor purity in LGG, which suggested that the expression of SMOC1 was more likely from tumor cells, which was consistent with a previous study (Brellier et al., 2011). The expression of SMOC1 was negatively correlated with levels of infiltrating B cells, CD8^+^ T cells, CD4^+^ T cells, macrophages, neutrophils, and dendritic cells, as well as gene markers of most immune cells in the LGG tumor microenvironment. Interestingly, there was no significant correlation between SMOC1 expression and tumor purity or most of the infiltrating immune cells in GBM. These results indicate that the expression of SMOC1 was significantly related to immune infiltrating cells in LGG. Together with the functional analysis, our findings strongly suggest that SMOC1 might play an important role in the tumor microenvironment of glioma, thereby influencing glioma development and progression. Further experimental studies are needed to validate our conclusions and explore the specific function of SMCO1 in glioma.

## Conclusion

In conclusion, our findings in this study suggest that SMOC1 was highly expressed in LGG, and the expression of SMOC1 was positively correlated with the survival of LGG patients. The functional analyses indicate that SMCO1 might play an important role in the glioma microenvironment, thereby influencing the development and progression of glioma. However, the prognostic value of SMOC1 in glioma needs to be validated in clinic samples, and as little was known about the biological function of SMOC1 in tumor genesis and progression, further *in vitro* and *in vivo* investigations are required to clarify the role of SMOC1 in glioma.

## Data Availability

The original contributions presented in the study are included in the article/Supplementary Material; further inquiries can be directed to the corresponding author.
