# Supplementary material for: Prognostic Potential of Secreted Modular Calcium-Binding Protein 1 in Low-Grade Glioma
Source: Front Mol Biosci. 2021 Nov 19;8:666623. doi: 10.3389/fmolb.2021.666623 (PMC8640086; doi:10.3389/fmolb.2021.666623)
Supplement: Supplementary file 3 [file Presentation1.PPTX]

## Slide 1
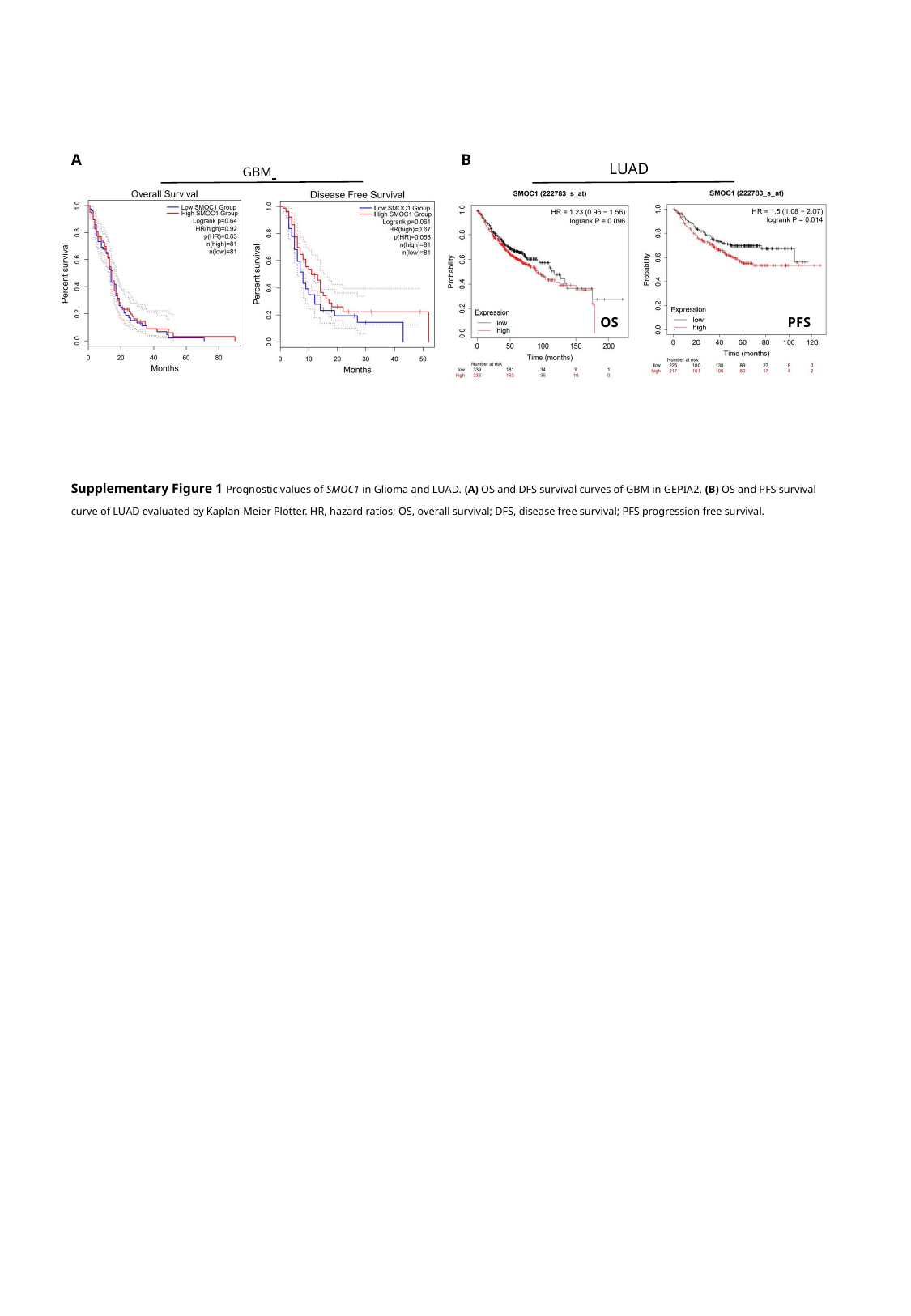

B
A
LUAD
GBM
OS
PFS
Supplementary Figure 1 Prognostic values of SMOC1 in Glioma and LUAD. (A) OS and DFS survival curves of GBM in GEPIA2. (B) OS and PFS survival curve of LUAD evaluated by Kaplan-Meier Plotter. HR, hazard ratios; OS, overall survival; DFS, disease free survival; PFS progression free survival.
