# Supplementary material for: Prognostic Potential of Secreted Modular Calcium-Binding Protein 1 in Low-Grade Glioma
Source: Front Mol Biosci. 2021 Nov 19;8:666623. doi: 10.3389/fmolb.2021.666623 (PMC8640086; doi:10.3389/fmolb.2021.666623)
Supplement: Supplementary file 5 [file Presentation2.PPTX]

## Slide 1
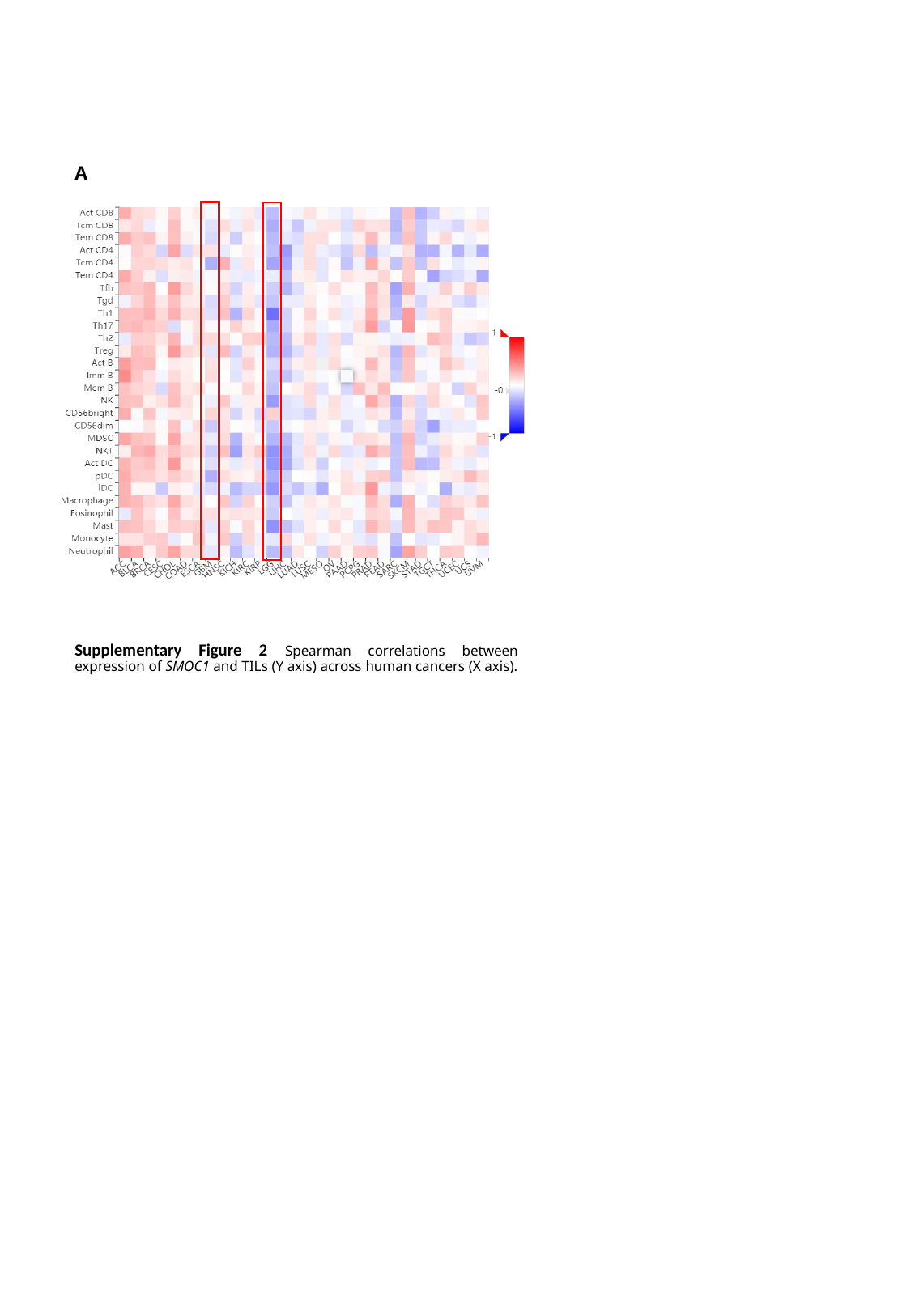

A
Supplementary Figure 2 Spearman correlations between expression of SMOC1 and TILs (Y axis) across human cancers (X axis).
